# Dietary acid load and risk of cardiovascular disease: a prospective population-based study

**DOI:** 10.1186/s12872-021-02243-8

**Published:** 2021-09-11

**Authors:** Parvin Mirmiran, Zeinab Houshialsadat, Zahra Bahadoran, Sajjad Khalili‑Moghadam, Mohammad Karim Shahrzad, Fereidoun Azizi

**Affiliations:** 1 Nutrition and Endocrine Research Center, Research Institute for Endocrine Sciences, Shahid Beheshti University of Medical Sciences, No. 24, Sahid-Erabi St, Yemen St, Chamran Exp, P.O.Box: 19395-4763, Tehran, Iran; 2 Shohada Tajrish Medical Center, Shahid Beheshti University of Medical Sciences, No. 24, Sahid-Erabi St, Yemen St, Chamran Exp, P.O.Box: 19395-4763, Tehran, Iran; 3 Endocrine Research Center, Research Institute for Endocrine Sciences, Shahid Beheshti University of Medical Sciences, Tehran, Iran

**Keywords:** Diet, Dietary acid load, Potential renal acid load, Net endogenous acid production, Cardiovascular disease

## Abstract

**Background and aim:**

Considering the inconsistencies in the cardiovascular effects of dietary acid load and the impact of dietary acidity on the acid–base homeostasis within the body, we aimed to assess the association of dietary acid load and the risk of cardiovascular disease (CVD) in a prospective community-based study.

**Materials and methods:**

Participants (n = 2369) free of CVD at baseline (2006–2008) were included from the Tehran Lipid and Glucose Study (TLGS) and followed up for a mean of 6.7 ± 1.4 years. Dietary intakes of the participants were assessed using a semi-quantitative food frequency questionnaire (FFQ). The dietary acid load was evaluated by Potential Renal Acid Load (PRAL) and Net Endogenous Acid Production (NEAP) scores. Both scores have used the macronutrient and micronutrient data of the Food Frequency Questionnaires. Multivariate Cox proportional hazard regression models were used to estimate the 6-years incident risk of CVDs across tertiles of PRAL and NEAP scores.

**Results:**

Mean age and body mass index of participants were 38.5 ± 13.3 years and 26.6 ± 4.8 kg/m^2^ at baseline. Within 6.7 ± 1.4 years of follow-up, 79 cases of cardiovascular events were reported. NEAP was significantly associated with the incidence of CVDs (HRs = 0.50, CI 0.32–0.96; *P* for trend = 0.032); however, after adjusting for potential confounders, no significant associations were observed between PRAL and NEAP scores and the risk of CVDs.

**Conclusions:**

This study failed to obtain independent associations between dietary acid load and the incidence of CVDs among an Asian population.

## Introduction

The acid–base balance within the body can be influenced by eating patterns and the acid load of the diet [1]. Animal-based food products raise the acidifying potentials of diets [2, 3] and negatively manipulate the metabolic and physiologic status [4]. The acidic dietary patterns have become prevalent within the global dietary transition [5], which may be a risk factor for the development of metabolic and cardiovascular diseases (CVDs).

Potential Renal Acid Load (PRAL) [6] and Net Endogenous Acid Production (NEAP) [7] are common and valid indicators of dietary acid load and overall nutritional quality of a diet [6, 7]. The PRAL score is comprised of dietary magnesium, potassium, phosphorus, calcium, and protein [7], and NEAP formula is based on dietary intake of protein and potassium [8]. Both scores are associated with the prevalence of type 2 diabetes [9, 10] and hypertension [11–14]. PRAL alone is linked to the incidence of insulin resistance [15, 16], metabolic syndrome [17], and progression of chronic kidney disease [18, 19].

Observational and epidemiological studies have reported inconsistent results on dietary acid load, and CVD outcomes [12, 14, 20, 21]. PRAL and CVD mortality were positively associated among Swedish individuals [22] and inversely related among the Japanese population [23]. In contrast, no significant association was detected between dietary acid load and CVD incidence among the Polish [24] and Dutch [12] populations. Meanwhile, the majority of the findings confirm the detrimental impacts of acidic dietary patterns on health [14, 17], which is mostly related to the increased tissue metabolic acidosis [25], changes in the glycemic [16, 26, 27] and lipid profiles [28] and, increased blood pressure [12, 13, 29].

Inadequate and inconsistent results bring unclear findings and challenge the documentation of standard global dietary guidelines. Here, we aimed to evaluate whether dietary acid load, defined as PRAL and NEAP scores, could be a predictor of CVD risk in the framework of a population-based study among an Asian population.

## Methods

### Study population

**Fig. 1:**
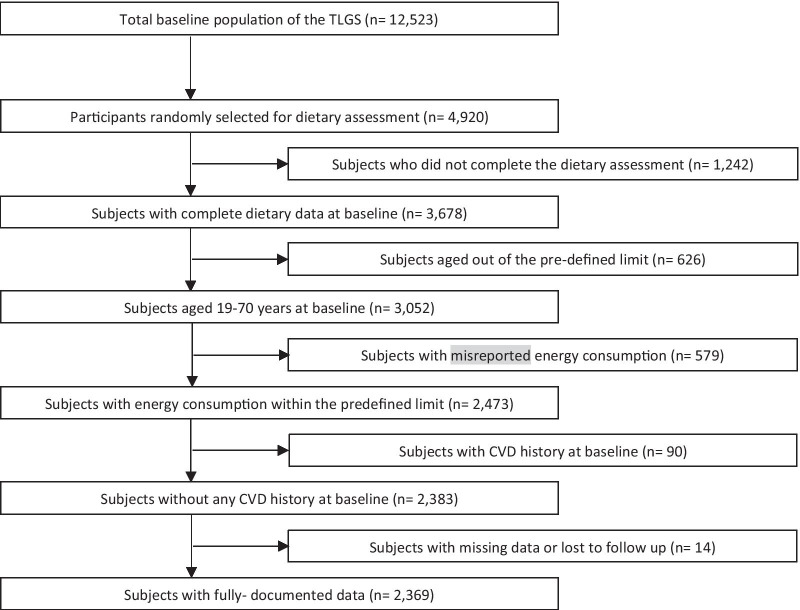
Flowchart of the study population

This study was conducted within the framework of the Tehran Lipid and Glucose Study (TLGS), a population-based study that aims to investigate non-communicable diseases (NCDs) within a representative sample of Iranians from district 13 of Tehran. The TLGS was initiated in 1999 and includes repeated measurements at 3-year intervals [30]. In total, 3678 men and women (aged ≥ 19) with complete demographic, anthropometric, biochemical, and dietary data, who have participated in the third TLGS examination (2006–2008), were recruited. Participants were excluded if they aged out of the predefined limit (70 < x < 19 years old; n = 626), and had misreported energy intake (4200 < x < 800 kcal/day; n = 579) and CVD history at baseline (myocardial infarction (MI), stroke, angina, coronary revascularization; n = 90). Participants were also excluded if they left the study within the follow-up period (n = 14). Finally, 2369 adults (1030 men and 1339 women) were included in the analyses (Fig. 1) [30, 31].

All participants have provided written consents at baseline. The study protocol complied with the 1975 Ethical Guidelines of the Helsinki Declaration and was approved by the Ethics Research Council of the Research Institute for Endocrine Science, Shahid Beheshti University of Medical Sciences.

### Demographic and anthropometric measurements

Trained interviewers have collected demographic information using pretested and standardized questionnaires [31]. Weight was recorded to the nearest 100 g by digital scales, while participants had light clothing. Height was measured to the nearest 0.5 cm, standing without shoes, using a drop-down tape-meter. Body mass index (BMI) was calculated by the division of weight (kg) by the square of height (m^2^). Waist circumference measurement was taken to the nearest 0.1 cm, midway between the lower border of the ribs and the iliac crest, while participants were minimally dressed by a soft measuring tape. Systolic (SBP) and diastolic blood pressures (DBP) were measured twice on the right arm using a standardized mercury sphygmomanometer; the mean of the two measurements was considered as the final blood pressure of the participants [31]. The frequency and duration of physical activity (expressed as metabolic equivalent hours per week; MET-h/wk) was assessed by a Modifiable Activity Questionnaire (MAQ) [32].

### Biochemical measurements

Baseline and follow-up blood samples were taken from all participants following a 12–14 h fasting. Triglyceride (TG) level was assayed by enzymatic colorimetric method with glycerol phosphate oxidase. Fasting serum glucose (FSG) was determined using enzymatic colorimetric analysis and glucose oxidase. High-density lipoprotein cholesterol (HDL-C) measurement was obtained after precipitation of the apolipoprotein-B-containing lipoproteins with phosphotungstic acid. The Pars Azmoon kits (Pars Azmoon Inc., Tehran, Iran) and Selectra 2 auto-analyzers (Vital Scientific, Spankeren, Netherlands) were used to perform the analyses.

### Dietary assessment

Demographic, dietary, anthropometric, and biochemical data were obtained from all participants at baseline (2006–2008). Trained interviewers used a 168-item semi-quantitative Food Frequency Questionnaire (FFQ) at the first examination to assess participants' dietary intake over the past year. The reliability, comparative validity and, stability of the questionnaire were previously evaluated in a random sample and proven to be reasonable [33].

The participant’s consumption frequency of each food item was recorded on a daily, weekly, or monthly basis [31], and the household-measured portion sizes were converted to grams. The energy and nutrient content analysis of raw food and beverages was based on the US Department of Agriculture Food Composition Table (USDA FCT). Since the Iranian Food Composition Table has limited nutritional data, it was only used for the traditional items not listed within the USDA FCT [34].

### Dietary acid load calculation

In this study, the dietary acid–base load was assessed by two indexes of PRAL and NEAP, using the following formula [7, 8]:

$$\begin{aligned} & {\text{PRAL }}\left( {{\text{mEq/d}}} \right) = \left[ {{\text{protein}}\left( {{\text{g/d}}} \right) \times 0.{49}} \right] \\ & \quad + \left[ {{\text{phosphorus}}\left( {{\text{mg/d}}} \right) \times 0.0{37}} \right] - \left[ {{\text{potassium}}\left( {{\text{mg/d}}} \right) \times 0.0{21}} \right] \\ & \quad - \left[ {{\text{calcium }}\left( {{\text{mg/d}}} \right) \times 0.0{13}} \right] - \left[ {{\text{magnesium }}\left( {{\text{mg/d}}} \right) \times 0.0{26}} \right] \\ \end{aligned}$$

$${\text{NEAP}}\left( {{\text{mEq/d}}} \right) = \left[ {{54}.{5} \times {\text{protein}}\left( {{\text{g/d}}} \right)/{\text{potassium }}\left( {{\text{mEq/d}}} \right)} \right]{-}{1}0.{2}$$

Dietary PRAL is a validated proxy for renal net acid excretion [1, 8], and the NEAP score is defined as the total nonvolatile acid load that results from endogenous acid production and gastrointestinal absorption [35]. A diet with acidifying potentials has higher PRAL and NEAP scores [4, 29]. There is a large variation in dietary acid load within the countries. The mean score of PRAL ranged from − 23.0 mEq/day in France [26] to − 22.0 mEq/day in Iran [18], − 21.8 mEq/day in Korea [14], − 14.6 mEq/day in Netherland [12], 10.4 mEq/day in Japan [11], and 22.1 mEq/day in China [37]. Likewise, mean dietary NEAP score ranged from 86.8 mEq/day in China [36], to 32.6 mEq/day among American children [6], and 31.5 mEq/day in France [26]. Both PRAL and NEAP were calculated using residual energy-adjusted nutrient intake data from the FFQs.

### Definition of terms

Diabetes was defined as FSG over 126 mg/dL, 2-h serum glucose above 200 mg/dL or use of anti-diabetic medications [37]. Hypertension was explained as SBP above 140 mm Hg, DBP higher or equal to 90 mm Hg, or concurrent use of antihypertensive medications [33].

### Definition of outcomes

Details of the collection of CVD-related data have been described elsewhere [31]. The CVD terminology was primarily defined as CHD-related events, stroke (a new neurological deficit that took ≥ 24 h), or CVD death (definite fatal stroke, definite fatal CHD, and definite fatal MI) [38]. CHD-related outcomes were including definite or probable MI, and angiographic-approved CHD [39].

In the current study, participants were followed up annually by telephone calls, and a trained nurse or a physician collected the required information on possible medical events. Further information was extracted from the medical records. The collected data were reviewed by an adjudication committee, which included a physician, an internist, an epidemiologist, a cardiologist, an endocrinologist, and associate external experts as needed. The final diagnosis was reported by a predefined coding protocol [40].

### Statistical analysis

In this study, IBM SPSS (SPSS Inc., Chicago, IL, USA, version 20.0) was used to perform the analyses, and *P*-values ≤ 0.05 were statistically significant. Mean (SD) values of the baseline characteristics of participants with and without CVD were compared by independent t-test. The chi-square test was used to compare frequencies (%) between two groups. Dietary intake of participants were compared across tertiles of PRAL and NEAP using analysis of variance (ANOVA) test. A univariate analysis was conducted for each potential confounder, and variables with *P*_E_ < 0.2 were included in the multivariable model; total dietary energy, and total dietary fat were included in the final model and the physical activity was eliminated. Cox proportional hazards regression models were used to evaluate the hazard ratios (HRs) and the 95% confidence intervals (CIs) of dietary acid load and CVD events, and person-year was considered as the underlying time metric. Time to event was defined as the time to the onset of an event, or time to the end of follow-up.

Three Cox proportional hazards regression models were identified across tertiles of PRAL and NEAP; model 1 was adjusted for sex, age and smoking status, and model 2 was further adjusted for energy intake (kcal/d) and total fat intake (g/d). The median value of each dietary tertile was used to assess the overall HR trends in the Cox proportional hazard regression model.

## Results

Mean age and BMI of participants were 38.5 ± 12.7 years and 26.6 ± 4.8 kg/m^2^ at baseline, respectively, and 43.5% were men. During an average follow-up period of 6.7 ± 1.4 years, 79 participants experienced CVD events (3.3%), and angiographic proven CVD, definite MI, unstable angina, and stroke were the most common outcomes.

Table 1 represents the distribution of major CVD risk factors and biochemical variables for participants with and without CVD events. Participants with CVD events were older (*P* = 0.001). Diabetes (13.2 *vs*. 3.7%, *P* = 0.001) and hypertension (42.1 *vs*. 9.4%, *P* = 0.001) were more prevalent among incident cases compared to the rest of the cohort. Also, a higher percentage of subjects with CVD events were current smokers (20.2 *vs*. 11.7%, *P* = 0.02).

**Table 1:** Baseline characteristics of the participants: Tehran Lipid and Glucose Study (TLGS)

	Participants with CVD outcomes (n = 79)	Participants without CVD outcomes (n = 2290)
Age (year)*	58.4 ± 9.7	37.4 ± 12.8
Male (%)*	68.4	42.6
Smoking (%)	20.2	11.7
Body mass index (m^2^/kg)	28.4 ± 4.4	26.5 ± 4.8
Waist circumference (cm)*	97.4 ± 9.9	87.9 ± 13.3
Serum creatinine (μmol/L)*	102 ± 24.1	91.8 ± 13.1
Systolic blood pressure (mm Hg)*	109 ± 14.8	109 ± 14.8
Diastolic blood pressure (mm Hg)*	79.9 ± 11.2	72.4 ± 10.3
Fasting blood glucose (mg/dL)*	104 ± 37.5	88.3 ± 16.1
Serum triglycerides (mg/dL)*	188 ± 102	132 ± 77.4
HDL-C (mg/dL)*	39.4 ± 7.6	43.3 ± 10.3
Diabetes (%)*	13.2	3.7
Hypertension (%)*	42.1	9.4

Independent *t* test was used for continuous variables, chi-square test was used for dichotomous variables.

Data are mean ± SD, unless stated otherwise

*P value ≥ 0.001

Dietary intakes of participants across tertiles of PRAL and NEAP are reported in Table 2. Mean dietary PRAL and NEAP were − 11.1 ± 18.6 mEq/day and 35.9 ± 10.9 mEq/day, respectively. Range of PRAL score across tertiles was < − 16.7 mEq/day, − 16.7 to − 2.45 mEq/day, and > − 2.45 mEq/day. Range of NEAP was < 30.6 mEq/day, 30.6–39.3 mEq/day, and > 39.3 mEq/day, in the first, second and third tertile, respectively. Participants in the lowest tertiles of PRAL had higher intakes of total dietary fiber, calcium, potassium, magnesium, potato, fruits and vegetables, and lower consumption of animal meat, cheese, grains and rice (*P* for all < 0.001). Similarly, lower NEAP score was related to lower consumptions of animal meat, cheese, grains and rice, and higher intakes of dietary fiber, calcium, potassium, magnesium, potato, and fruits and vegetables (*P* for all < 0.001).

**Table 2:** Dietary intake of participants across tertiles of PRAL and NEAP: Tehran Lipid and Glucose Study (TLGS)

	PRAL (mEq/day)			NEAP (mEq/day)		
	< − 16.7 (n = 789)	− 16.7 to − 2.45 (n = 790)	> − 2.45 (n = 790)	< 30.6 (n = 789)	30.6 to 39.3 (n = 794)	> 39.3 (n = 786)
	− 31.6 ± 13.0	− 9.49 ± 4.07	7.76 ± 8.6	24.9 ± 4.11	34.6 ± 2.47	48.3 ± 7.75
*Nutrient intake*						
Energy intake (kcal)	2371 ± 23.7	2150 ± 25.1	2260 ± 27.4*	2196 ± 23.8	2269 ± 24.9	2316 ± 27.8*
Protein (% of energy)	13.4 ± 0.08	13.3 ± 0.08	14.1 ± 0.08*	13.11 ± 0.08	13.6 ± 0.08	14.2 ± 0.08*
Carbohydrate (% of energy)	58.7 ± 0.25	56.8 ± 0.25	56.1 ± 0.25*	58.8 ± 0.25	56.6 ± 0.25	56.3 ± 0.25**
Fat (% of energy)	31.3 ± 0.25	32.2 ± 0.25	31.2 ± 0.25**	31.6 ± 0.25	32.1 ± 0.25	31.0 ± 0.25*
Fiber (gr/d)	40.4 ± 0.59	35.9 ± 0.59	34.1 ± 0.58*	39.7 ± 0.59	35.6 ± 0.59	35.4 ± 0.59*
Calcium (mg/d)	1362 ± 14.5	1229 ± 14.4	1087 ± 14.4*	1364 ± 14.3	1247 ± 14.2	1067 ± 14.3*
Potassium (mg/d)	4476 ± 29.0	3655 ± 29.0	2936 ± 28.9*	4480 ± 28.8	3659 ± 28.7	2925 ± 28.8*
Magnesium (mg/d)	397 ± 2.83	366 ± 2.82	346 ± 2.81*	398 ± 2.81	366 ± 2.80	345 ± 2.81*
phosphorus (mg/d)	1448 ± 10.8	1438 ± 10.7	1445 ± 10.7*	1445 ± 10.7	1462 ± 10.7	1424 ± 10.7*
*Food intake*						
Meat (gr/d)	47.5 ± 1.46	50.9 ± 1.46	63.2 ± 1.45*	42.5 ± 1.43	52.1 ± 1.42	67.1 ± 1.43*
Grains (gr/d)	319 ± 5.91	389 ± 5.91	486 ± 5.88*	323 ± 5.92	387 ± 5.90	485 ± 5.93*
egg (gr/d)	14.4 ± 0.44	13.8 ± 0.44	15.9 ± 0.44*	14.1 ± 0.44	14.1 ± 0.44	15.1 ± 0.44*
cheese (gr/d)	19.0 ± 0.72	18.9 ± 0.72	20.3 ± 0.72*	18.5 ± 0.72	19.1 ± 0.71	20.6 ± 0.72*
Fish (gr/d)	6.27 ± 0.74	7.84 ± 0.74	6.46 ± 0.74*	6.53 ± 0.74	6.07 ± 0.74	7.97 ± 0.75*
Rice (gr/d)	202 ± 5.85	248 ± 5.85	307 ± 5.79*	208 ± 5.84	248 ± 5.82	302 ± 5.85*
Coffee (ml/w)	59.4 ± 77.6	58.8 ± 68.3	57.1 ± 65.2**	55.3 ± 73.3	61.6 ± 70.6	58.4 ± 70.5**
Fruit and vegetable (gr/d)	732 ± 8.61	497 ± 8.61	303 ± 8.57*	734 ± 8.60	490 ± 8.56	307 ± 8.61*
Potato (gr/d)	19.9 ± 0.69	17.0 ± 0.69	14.2 ± 0.69*	18.9 ± 0.69	17.9 ± 0.69	14.4 ± 0.70*
Legumes (gr/d)	17.1 ± 0.76	15.8 ± 0.77	14.9 ± 0.76*	16.1 ± 0.76	16.1 ± 0.76	15.5 ± 0.76*

Analysis of variance (ANOVA) was done and the first tertile was considered as the reference

*PRAL* potential renal acid load, *NEAP* net endogenous acid production

Data are presented as mean ± SE or percentage, unless stated otherwise

Adjusted for energy intake

*P value < 0.001

**P value < 0.05

The hazard ratio (95% CI) of CVD incidence across tertile categories of PRAL and NEAP are shown in Table 3. The risk of CVD events was reduced significantly in the NEAP crude model (HRs = 0.50; CI 0.32–0.96; *P* trend = 0. 032). No significant associations were observed for PRAL and NEAP scores and CVD incidence after adjusting for age, sex, and smoking status in the second model, and total energy and total fat intake in the third model.

**Table 3:** Hazard ratio (95% CI) of cardiovascular disease across tertiles of PRAL and NEAP: Tehran Lipid and Glucose Study (TLGS)

	T1	T2	T3	*P* trend
	< − 16.7 (n = 789)	− 16.7 to − 2.45 (n = 790)	> − 2.45 (n = 790)	
PRAL (mEq/day)				
Crude	1	0.67 (0.39–1.14)	0.65 (0.38–1.11)	0.094
Model 1	1	0.73 (0.43–1.24)	0.79 (0.46–1.36)	0.346
Model 2	1	0.75 (0.44–1.28)	0.80 (0.46–1.37)	0.367

	T1	T2	T3	*P* trend
	< 30.6 (n = 789)	30.6 to 39.3 (n = 794)	> 39.3 (n = 786)	
NEAP (mEq/day)				
Crude	1	0.63 (0.37–1.07)	0.50 (0.32–0.96)	0.032
Model 1	1	0.72 (0.42–1.21)	0.76 (0.44–1.33)	0.986
Model 2	1	0.73 (0.43–1.23)	0.76 (0.44–1.32)	0.988

Cox proportional hazard regression model was used to estimate hazard ratio (HR) and 95% confidence intervals (CI) for cardiovascular disease across tertiles of dietary acid load

PRAL, potential renal acid load; NEAP, net endogenous acid production

Model 1: adjusted for sex, age, smoking status

Model 2: adjusted for sex, age, smoking status, dietary energy intake (kcal/d), total fat intake (g/d)

## Discussion

In this population-based cohort study, we assessed the potential associations between dietary acid load and CVD outcomes. After adjusting for potential confounders, no significant associations were observed for PRAL and NEAP and CVD incidence risk, which may be explained by the low number of CVD cases, relatively short follow-up period, young study population, and potential changes in the dietary patterns of participants over time.

Our findings, however, were in line with the results of a large-scale study in Poland [21]. In contrast, a 2016 cross-sectional study on Korea National Health and Nutrition Examination data reported a positive association between the dietary acid load and incidence risk of CVD [14]. The higher dietary acid load has also elevated the risk of CVD mortality among Japanese individuals [23], increased 10-year mortality of patients with coronary artery bypass surgery in Iran [41] and, influenced the likelihood of chronic peripheral arterial disease among Americans [42]. Indeed, various populations differ in baseline characteristics and habitual dietary intakes, composing a dietary acid load spectrum [11, 21].

Previous publications have reported associations between the dietary acid load and CVD risk factors [11, 29, 43–46]. Dietary acidity induces low-grade acidosis that is linked to the development of metabolic complications, including diabetes [47], hypertension, and renal and bone complications [19, 48, 49]. PRAL and NEAP scores were both positively associated with serum TG levels [43]. PRAL was also independently associated with increased TG, SBP [44], and low-density lipoprotein cholesterol (LDL) [11] levels, and inversely related to fasting blood glucose [44]. In a 2018 systematic review and meta-analysis, a non-linear association was observed between NEAP and hypertension, and a 20-unit increase in PRAL value raised the risk of hypertension by 3% [45]. Furthermore, one study in South China highlighted the gender-dependent hypotensive properties of PRAL, which appeared insignificant in the context of NEAP [29]. A recent meta-analysis found positive associations between PRAL scores and SBP, DBP, insulin concentrations, and diabetes [50]. In contrast, no cross-sectional or longitudinal associations were observed between dietary acid load and various blood pressure indices in Swedish middle-aged men [51], metabolic syndrome risk factors [52], and risk of hypertension in older Dutch adults [12].

The mean dietary PRAL and NEAP in this study were − 11.1 ± 18.6 mEq/day and 35.9 ± 10.9 mEq/day, respectively, which confirms the dietary pattern of our population to be less acidic comparing to the Korean (PRAL: − 21.8 mEq/day) [14] and French (PRAL: − 23.0 mEq/day) [26] populations. Nevertheless, the dietary patterns of Chinese (PRAL: 22.1 mEq/day) [29] and Japanese (10.4 mEq/day) populations [11] appeared to be more alkalizing. This study was conducted on Iranian adults with transitional dietary patterns and an estimated animal-to-plant protein ratio of approximately 1.3–2.1.4 [53].

Participants with lower values of PRAL and NEAP scores had lower intakes of animal products and higher intakes of fruits and vegetables. It is generally confirmed that animal-based food items hold acidifying properties [3], whereas plant-food sources are more alkalizing [54]. Western dietary patterns, with an average 15–17% of energy from animal protein, are major acid suppliers to the body [2, 3]. On the contrary, the Dietary Approach to Stop Hypertension (DASH) pattern that is mainly comprised of plant foods and monounsaturated and polyunsaturated fats substantially reduces the dietary acid load (NEAP; 31 mEq/day vs. 78 mEq/day) [55]. Inadequate consumption of low-potassium fruit and vegetables in large samples of American individuals had adverse effects on the dietary acid load [3]. The dietary potassium of vegetables can bind to organic anions and metabolize to bicarbonate, which is ultimately capable of reducing NEAP [46]. The total or partial replacement of low-nutrient and energy-dense food items with fruits and vegetables can reduce the overall NEAP regardless of the amount of protein required [56].

To date, mechanistic information linking dietary acid load and CVD outcomes is mostly attributed to the role of chronic metabolic acidosis and hypertension. High adherence to Western dietary patterns enhances metabolic acidosis, and in return, increases the production of cortisol, ammoniagenesis, and renal acid excretion [6, 57]. Together, this leads to the diagnosis of hypertension [6]. In addition, restrictions in the dietary intake of potassium can affect the vascular vasodilation and damage blood vessels, and result in intracellular potassium deficiencies and compensatory sodium gains into the cell for the maintenance of the tonicity and volume [57]. Other mediators of metabolic acidosis and hypertension are the reduced excretion of citrate, increased release of calcium and cortisol, and the quality and quantity of the dietary protein [48]. High dietary acid load and chronic metabolic acidosis are also closely linked to the reduced affinity of the insulin to its receptor, increased risk of insulin resistance, and subsequently, hyperglycemia [15–17]. CVD can be autonomously promoted from insulin resistance through various pathways, including coronary microcirculatory dysfunction [58] and increased arrhythmogenesis [59].

This study has a number of strengths, including the high follow-up rate in the framework of a prospective population-based design, and the use of a validated FFQ for the assessment of habitual dietary intakes. The use of dietary PRAL for the measurement of dietary acid–base balance is one of the main limitations of this study. Although PRAL and NEAP scores have been widely used in previous publications, they are measured indirectly from the FFQs and can be influenced by inaccurate dietary reports [6, 7]. Also, the variations in the dietary patterns over time, the actual nutrient composition of specific meals, the preparation methods, and the nutrients’ absorption within the gastrointestinal tract are not considered by the PRAL and NEAP equations. Moreover, the low rate of CVD events in our population could have led to underestimations in CVD incidence. Lastly, the relatively short follow-up period with a relatively young population made it difficult to follow CVD endpoints.

## Conclusion

Our results did not show any significant associations between dietary acid load and risk of CVDs within a representative sample of Iranians. The global growth in CVDs prevalence, the high treatment costs and burden of CVDs, and the critical role of diet in cardiovascular health call for investigations on dietary acid load and CVDs risk, with larger-scale samples and longer follow-up durations.

## Data Availability

The database used and/ or analyzed during the current study available from the corresponding author on reasonable request.

## Abbreviations

BMI: Body mass index
CHD: Coronary heart disease
CVD: Cardiovascular disease
DBP: Diastolic blood pressure
FFQ: Food frequency questionnaire
FSG: Fasting serum glucose
HDL-C: High-density lipoprotein cholesterol
LDL: Low-density lipoprotein cholesterol
NCDs: Non-communicable diseases
NEAP: Net endogenous acid production
PRAL: Potential renal acid load
SBP: Systolic blood pressure
TC: Total cholesterol
TG: Triglyceride
TLGS: Tehran lipid and glucose study
